# Room-temperature synthesis of earth-abundant semiconductor ZnSiN_2_ on amorphous carbon

**DOI:** 10.1038/s41598-021-82845-6

**Published:** 2021-02-05

**Authors:** Horácio Coelho-Júnior, Bruno G. Silva, Cilene Labre, Renan P. Loreto, Rubem L. Sommer

**Affiliations:** Brazilian Center for Physics Research, 22.290-180, Rio de Janeiro, RJ Brazil

**Keywords:** Semiconductors, Synthesis and processing

## Abstract

This manuscript reports room-temperature one-step synthesis of earth-abundant semiconductor ZnSiN_2_ on amorphous carbon substrates using radio frequency reactive magnetron co-sputtering. Transmission Electron Microscopy and Rutherford Backscattering Spectrometry analysis demonstrated that the synthesis has occurred as ZnSiN_2_ nanocrystals in the orthorhombic phase, uniformly distributed on amorphous carbon. The technique of large-area deposition on an amorphous substrate can be interesting for flexible electronics technologies. Our results open possibilities for environmentally friendly semiconductor devices, leading to the development of greener technologies.

## Introduction

Semiconductor materials can be considered one of the technology pillars of contemporaneous life. A great amount of work in semiconductor basic and applied science^1^ has been done in the past years. In particular, nitrogen-based semiconductors revolutionized the technology of light-emitting devices^2–4^. In addition, the technological integration of those nitrides combined with semiconductor materials already used in industry is promising for manufacturing systems with multiple functionalities. Gallium nitride (GaN) synthesized by N implantation into gallium arsenide (GaAs), for example, is important for microelectronics applications^5–7^.

Synthesis of eco-friendly materials is within one of the fundamental principles of green nanotechnology^8–12^, which is a strong demand for a post-modern society^13^, and has high socioeconomic status worldwide^14–16^. Studies on green technologies are quite recent^17^ and take into consideration the long-term demand of elements^18^ and its environmental impacts in the near future^19,20^. Exploring these aspects, scientists also have paid attention to abundance^21^ and toxicity of elements for materials synthesis, aiming various applications^10,22–25^. Recently, studies based on computational screening followed by high-pressure synthesis, reported the discovery of a class of nitride semiconductors composed of earth-abundant elements^26^. In particular, zinc silicon nitride (ZnSiN_2_) is a member of the ternary zinc nitride wide band gap semiconductors family^27,28^. Researchers have categorized ZnSiN_2_ within the emerging materials class^29^ as a potential candidate for photovoltaic absorber^30^ and endorsed it for technological integration^31–33^. ZnSiN_2_ synthesized by an ammonothermal approach crystallize in the orthorhombic phase and has the lattice parameters a = 5.25 Å, b = 6.28 Å and c = 5.02 Å, with a band gap of 3.7 eV at room-temperature^27^. It is important to emphasize that ZnSiN_2_ synthesis has only a few reports in the Inorganic Crystal Structure Database to date (ICSD codes #200584^27^ and #656276^28^).

In this work, we report room-temperature one-step synthesis of earth-abundant and non-toxic semiconductor ZnSiN_2_ on amorphous carbon by using radio frequency (RF) reactive magnetron co-sputtering. The co-sputtering technique can also be suitable for dopant studies^32,34^ and thus favorable to diluted magnetic semiconductors (DMS) synthesis aiming possible applications in spintronics^33,35^. Magnetron sputtering is largely applied for the deposition of a wide range of thin film materials^36,37^ and it is also applicable in greener synthesis strategies^38^. Additionally, magnetic materials synthesis at room-temperature on amorphous substrates reveals a perspective for the development of flexible spintronics^39^. Our synthesis brings new perspectives to synthesize ZnSiN_2_ without the need for expensive or complex substrate preparation or thermal treatment process. This process also has the advantage to allow large-scale/large-area synthesis, even on an amorphous substrate, a strong point to applications also in macro electronics^40,41^ taking into account environmentally friendly concepts^42^.

## Results and discussion

**Figure 1:**
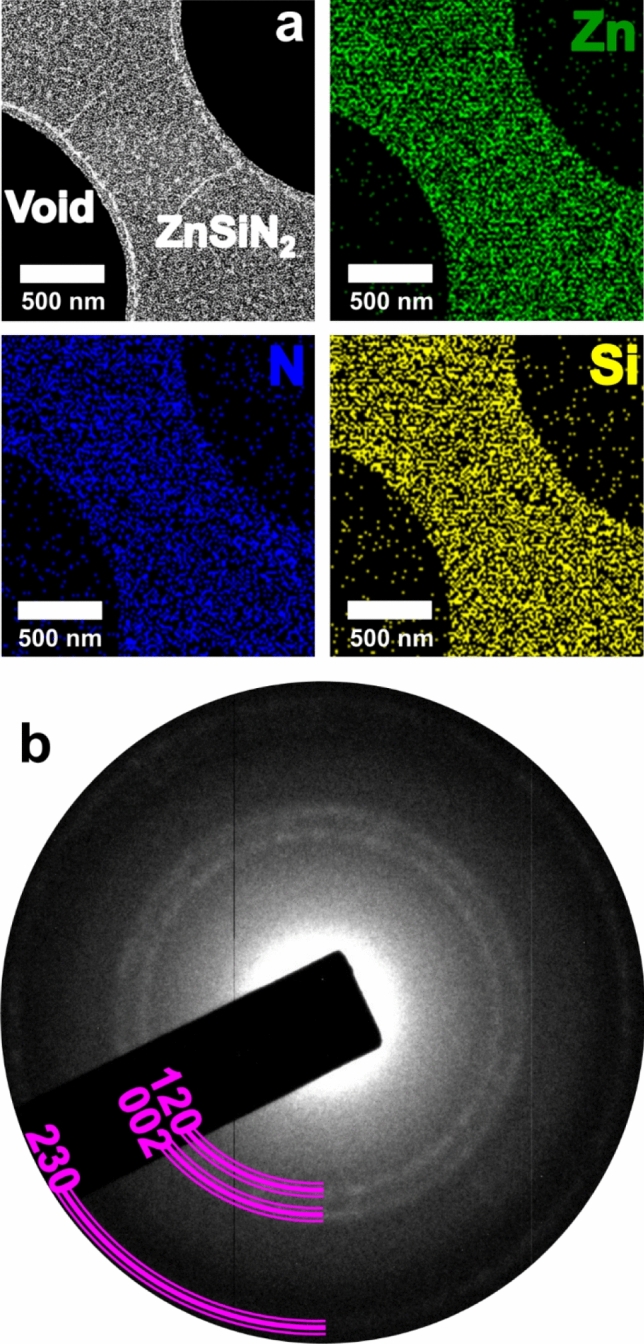
(**a**) ADF-STEM image and a set of EDS maps of a region with deposited ZnSiN_2_, showing the presence of Zn (green), Si (yellow) and N (blue). (**b**) SAED pattern with the correspondent diffraction planes.

In Fig. 1a, we present an ADF-STEM (Annular Dark Field-Scanning Transmission Electron Microscopy) micrograph, where is possible to observe a bright contrast corresponding to the region with the synthesized compound. This contrast comes from the nanostructures examined, which appear as bright small dots. We would like to emphasize that ADF-STEM mode was intentionally applied to maximize contrast-diffraction effects, which is possible because those nanostructures have a clear crystalline character. It is possible to observe that the bright dots are evenly distributed on the amorphous carbon area. Finally, a set of EDS (Energy Dispersive X-rays Spectroscopy) maps of ZnSiN_2_ demonstrates the uniform distribution of nanostructures on amorphous carbon, in corroboration with the ADF-STEM micrograph. Figure 1b shows a SAED (Selected Area Electron Diffraction) pattern of ZnSiN_2_ with an annular pattern originated from nanocrystals and the amorphous substrate. Those rings are highlighted with a violet semicircle with the corresponding ZnSiN_2_ family of planes indexed as (120), (002), and (230) of the orthorhombic phase, according to theoretical calculations for this material^27^.

**Figure 2:**
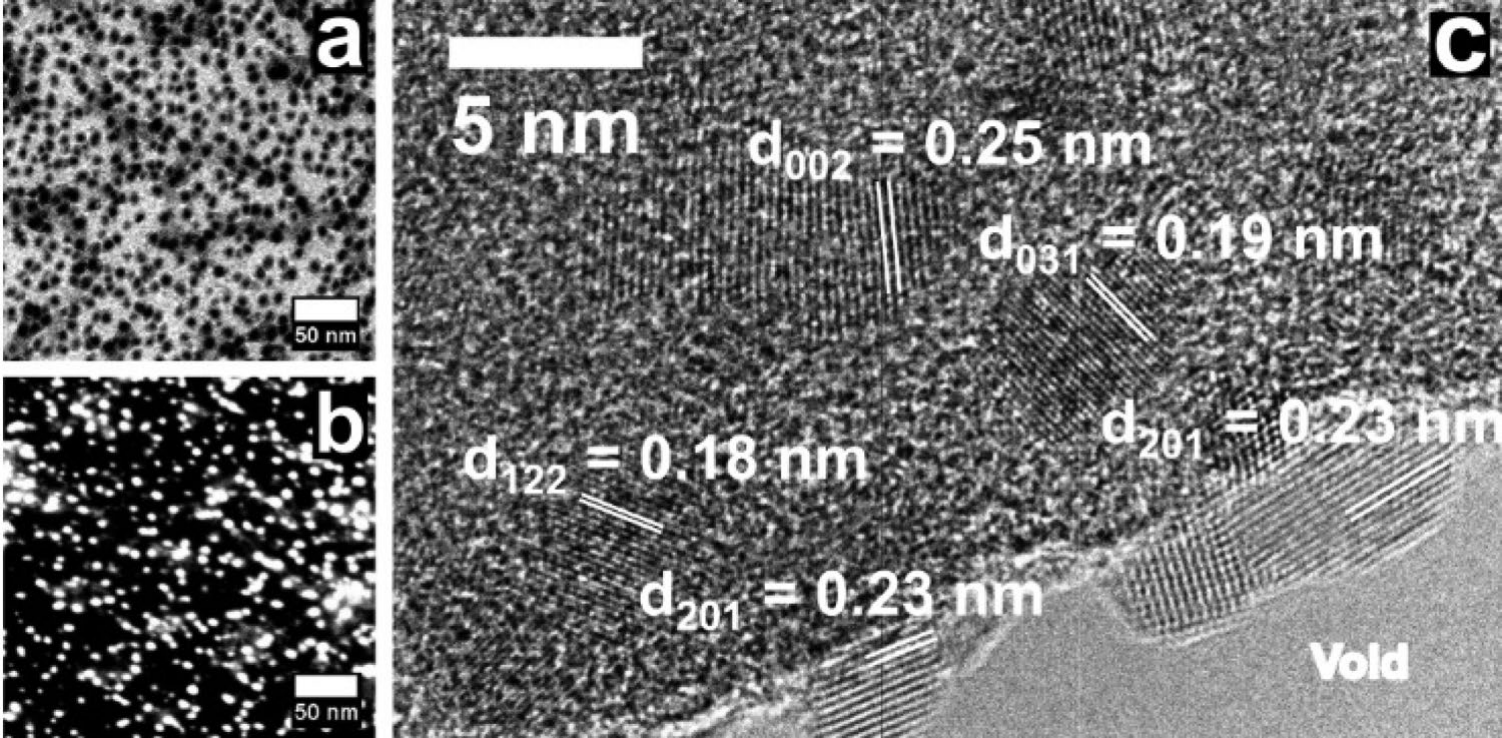
(**a**) ABF-STEM, (**b**) HAADF-STEM and (**c**) HRTEM micrographs of ZnSiN_2_ nanostructures with their respective lattice spacing in its orthorhombic phase.

In Fig. 2 we detail the nanostructures presented in Fig. 1 using high magnifications. Figure 2a presents an ABF-STEM (Annular Bright Field-Scanning Transmission Electron Microscopy) micrograph showing the distribution and morphology of ZnSiN_2_ nanostructures. The bright small dots viewed in Fig. 1a now can be seen as dark dots and demonstrate the uniform distribution of the nanostructures on amorphous carbon, reinforcing the synthesis capability by using sputtering deposition. Besides, the nanostructure sizes are practically uniform, indicating the potential for nucleation since the initial synthesis at room-temperature. This characteristic can be useful, for example, in 2D materials synthesis aiming photocatalyst applications as done in the work from Bai et al.^43^. In this study, the authors explain that ZnSiN_2_ can have a higher band gap with respect to other zinc nitrides (like ZnGeN_2_ and ZnSnN_2_), being proposed as efficient photocatalysts for water splitting. According to the first perspective, we believe that our work opens the possibility of manufacturing devices like thin film transistor (TFT) due to the potential production of single layers with a wide band gap in a large-scale/large-area with good uniformity.

Figure 2b shows the HAADF-STEM (High-Angle Annular Dark Field) image of ZnSiN_2_ in corroboration to Fig. 1a. Here we aim to explore variations in atomic number from elements in the sample. In this micrograph, it is possible to observe that the nanocrystals are displayed in higher contrast, because of the reduction of the contribution from amorphous carbon in the image formation. The nanocrystals observed in Fig. 1a are in agreement with HAADF measurement in Fig. 2b which show that ZnSiN_2_ synthesis has occurred effectively. In Fig. 2c is possible to observe the interplanar spacing of ZnSiN_2_ nanostructures distributed on amorphous carbon. This HRTEM (High-Resolution Transmission Electron Microscopy) micrograph was obtained in the region between ZnSiN_2_/amorphous-carbon and the void, displaying the interplanar spacing that matches the crystallographic planes of the orthorhombic phase of ZnSiN_2_^27^ also in accordance with Fig. 1b.

**Figure 3:**
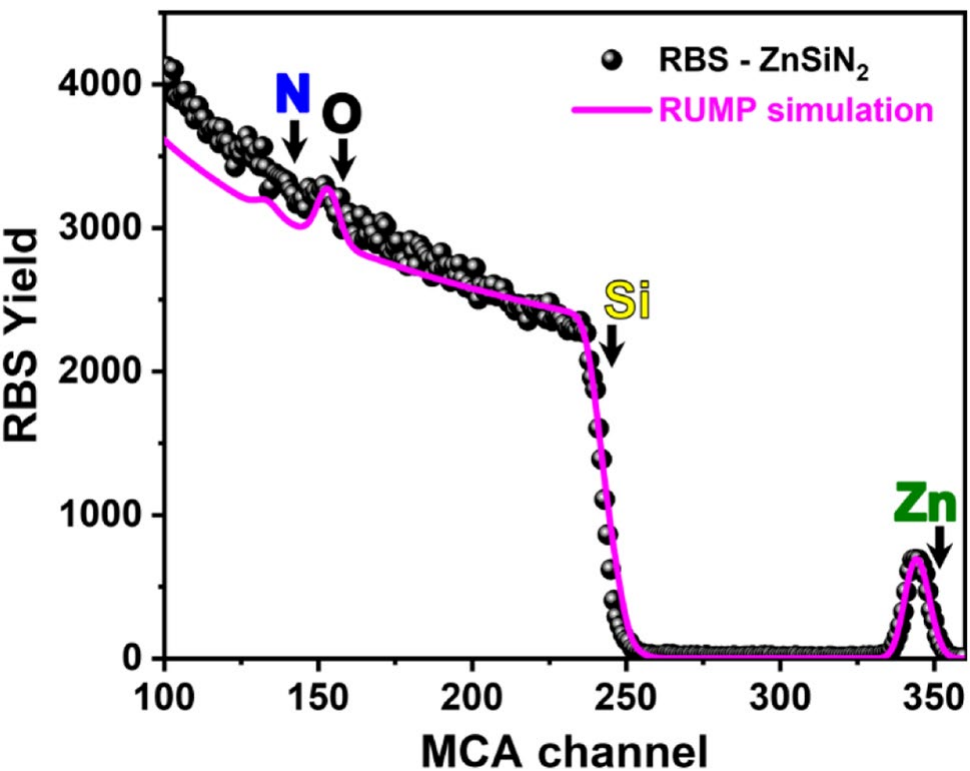
RBS spectrum of ZnSiN_2_. Full circles correspond to measured RBS spectrum and line corresponds to the RBS simulation performed by RUMP code^44^.

**Figure 4:**
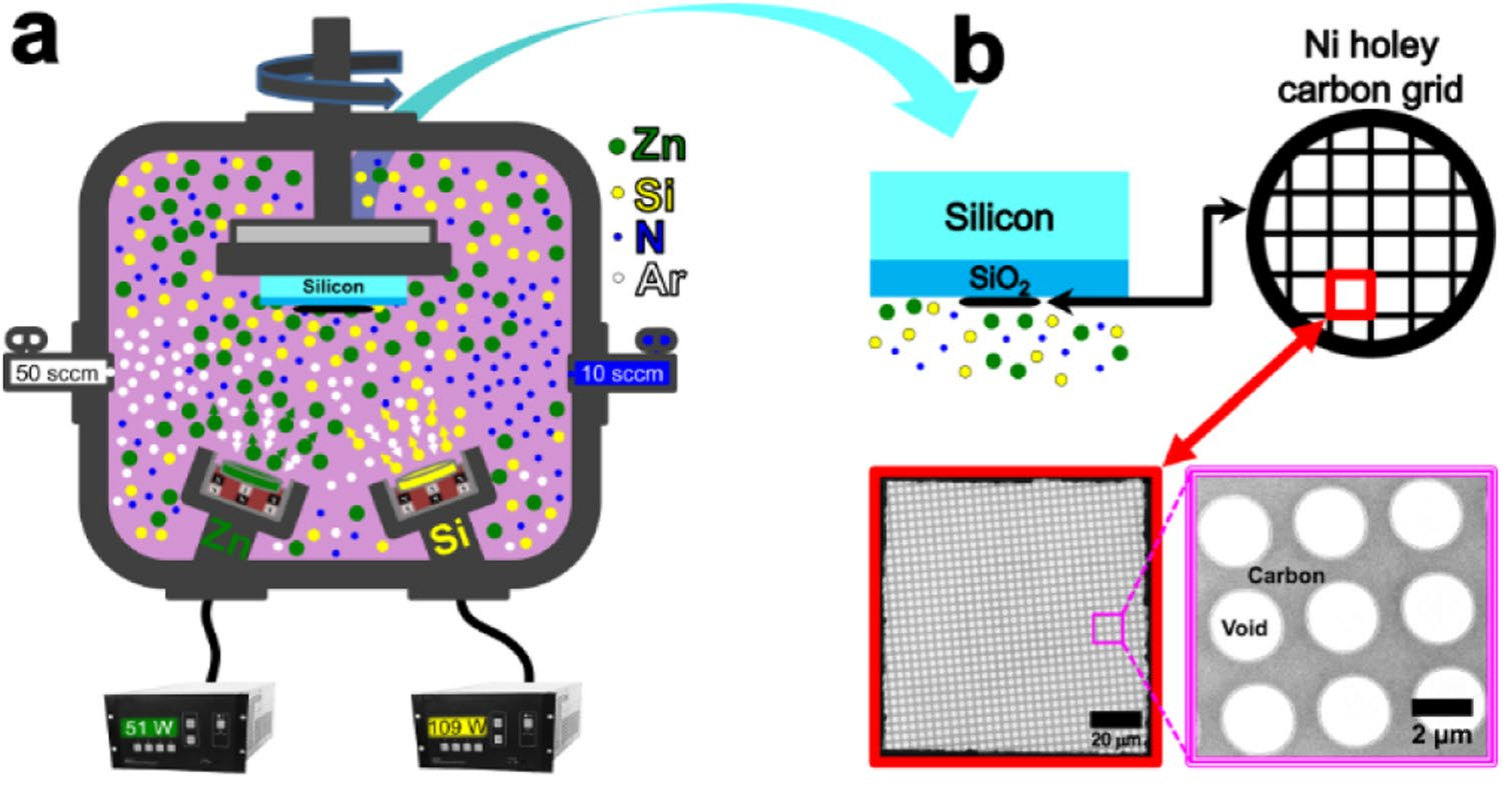
(**a**) Schematic setup of combinatorial sputtering for ZnSiN_2_ synthesis on amorphous carbon. (**b**) The red square indicates a zoom from one grid quadrant while the magenta square shows a region containing amorphous carbon with circular voids.

In addition to TEM measurements, we present our quantitative analysis using RBS (Rutherford Backscattering Spectrometry). Figure 3 shows the RBS spectrum of ZnSiN_2_ and its RBS simulation performed by the RUMP code^44^. The RBS measurement was obtained from ZnSiN_2_ synthesized on SiO_2_/Si (see Fig. 4b) complementing the qualitative EDS analysis (Fig. 1). It is possible to observe that the edge from Zn, Si, and N signals are well defined in the RBS spectrum of Fig. 3, which corroborates the presence of those elements in our synthesized material. The signals from the backscattered ^4^He^++^ from Zn appear at around channel 344 while from Si is pronounced like a single edge around channel 242 and from N is smoothly delineated around channel 142. The RBS signal around channel 156 is originated from oxygen in the SiO_2_/Si substrate. We use a layered structure in order to effectively simulate the experimental spectrum of ZnSiN_2_: layer 1: 7 nm of ZnSiN_2_ layer composition; layer 2: 25 nm of SiO_2_; layer 3: silicon substrate. This layered model is suitable to simulate the Zn and Si signals in the RBS spectrum, indicating the good quality of our fit. Once the sputtering technique promotes a uniform synthesis of the ZnSiN_2_ on amorphous carbon (as demonstrated in Fig. 1), it is reasonable to expect that the synthesized ZnSiN_2_ is also evenly distributed on SiO_2_/Si. Therefore, we have demonstrated that our synthesis was successfully also in both, large-area amorphous carbon and large-area silicon oxide. It is important to point out that this work shows that we were able to promote a large-area of material synthesis through a single-step technique at room temperature, features that can be of interest for TFT technologies. In this context, is important to notice that the manufacturing of flexible TFT at room temperature using amorphous oxide semiconductors is already a reality^45^. In reference^45^ the authors expressed the importance of TFT fabrication at room temperature, as well as the production in large-area for the development of flexible electronic devices. Furthermore, flexible and freestanding single layers zinc-based semiconductors, produced in large areas have been promising to enhance solar water-splitting efficiency^46^, as well as for other photovoltaic applications^47,48^.

## Conclusion

In summary, we report room temperature one-step synthesis of earth-abundant and non-toxic semiconductor ZnSiN_2_ on amorphous carbon substrates by using radio frequency reactive magnetron co-sputtering. The synthesis occurred as nanocrystals of ZnSiN_2_ in its orthorhombic phase. The synthesis technique demonstrates to be capable to produce ZnSiN_2_ in large-scale/large-area on amorphous substrates while taking into account greener concepts applied to advanced materials for flexible electronics.

## Experimental section

### Synthesis

ZnSiN_2_ was synthesized by sputtering using Zn and Si targets (both with 99.99% purity) simultaneously under a mixed Ar (50 sccm) and N_2_ (10 sccm) atmosphere for a nominal chamber pressure of 5 mTorr. The system base pressure was 1 × 10^–7^ Torr. Figure 4a shows the schematic setup of the synthesis chamber, where Zn and Si targets were kept under RF power of 51 W and 109 W, respectively. A Ni holey carbon grid (Quantifoil Q25035 R2/1 200 M) usually applied in TEM experiments was fixed on a SiO_2_/silicon substrate maintained at room temperature and in constant rotation during the synthesis. Figure 4b shows the grid/substrate accommodated on the holder, highlighting the grid (~ 3 mm diameter) separately, showing the morphology of the carbon amorphous region where the ZnSiN_2_ synthesis will be held. Before synthesis, the silicon substrate was cleaned with acetone, isopropyl alcohol, and deionized water. The carbon grid was used for TEM experiments. The material deposited on SiO_2_/Si was submitted to RBS measurement.

### Characterization

A JEOL FEG JEM 2100F transmission electron microscope (TEM) operated at 200-kV of acceleration voltage and equipped with an energy-dispersive x-ray spectrometer (EDS-Noran Seven) was used for HRTEM, SAED, and EDS maps. The STEM mode images were obtained using an annular dark-field (ADF) and annular bright-field (ABF) detectors. EDS measurements were performed in STEM mode. RBS was employed to evaluate the overall composition of the synthesized sample. It was carried out by using a 1.2-MeV ^4^He^++^ ion beam produced by the 3-MV Tandetron accelerator from High Voltage Engineering Europa of the Ion Implantation Laboratory at Universidade Federal do Rio Grande do Sul (UFRGS), Brazil.
